# Electrochemical Activity of Lignin Based Composite Membranes

**DOI:** 10.3390/polym13040643

**Published:** 2021-02-21

**Authors:** Marya Baloch, Mikel Alberro, Jalel Labidi

**Affiliations:** 1 Department of Chemical and Environmental Engineering, University of the Basque Country (UPV/EHU), Plaza Europa 1, 20018 Donostia-San Sebastian, Spain; marya.baloch@ehu.eus; 2 Department of Electronic Technology, University of the Basque Country (UPV/EHU), Plaza Europa 1, 20018 Donostia-San Sebastian, Spain; mikel.alberro@ehu.eus

**Keywords:** batteries, lignin, inexpensive, environmentally friendly, quinone, redox activity

## Abstract

Our society’s most pressing challenges, like high CO_2_ emission and the constant battle against energy poverty, require a clean and easier solution to store and utilize the renewable energy resources. However, recent electrochemical components are expensive and harmful to the environment, which restricts their widespread deployment. This study proposes an easy method to synthesize and fabricate composite membranes with abundantly found biomass lignin polymer to replace conventional costly and toxic electrode materials. Easier manipulation of lignin within the polymeric matrix could provide the improved composite to enhance its electrochemical activity. Our major focus is to activate the quinone moiety via oxidation in the polymeric mixture using a strong ionic acid. The physico-chemical and electrochemical characterizations of two different lignins within varied polymeric mixture compositions have been carried out to confirm that the redox properties of pure unmodified lignin could be achieved via intrinsic mutual sharing of the structural properties and intercross linkage leading to improved integrity and redox activity/conductivity.

## 1. Introduction

Electric energy storage systems are widely demanded by various sectors both for their great versatility and advantages as environmentally sustainable alternatives such as storage of energy from renewable resources [1,2]. With a growing population, the CO_2_ emissions from internal combustion engines are increasing with a terrible effect both on personal health and the environment. From electrochemical energy storage, batteries are the systems with the highest storage capacity, due to their high energy density [3,4,5]. Although batteries are efficient, convenient, reliable, and easy to use, their useful life and autonomy are limited, in addition to their doubtful sustainability in terms of materials, since the materials used (both metals and non-metals) can generate various polluting constituents during the process [6]. Therefore, the practicality of these inorganic materials is limited by various factors such as scarcity, high cost, toxicity, and difficulty of processing techniques. It is essential to find a replacement among materials that are abundant and sustainable, i.e., organic polymers.

Redox polymers have gained popularity as a replacement for harmful battery components due to their non-toxic nature and enhanced electrical properties such as poly (3,4-ethylenedioxyphene) (PEDOT) [7], polyaniline (PANI) [8], polythiophene (PTh) [9], polypyrrol (PPy) [10], polyacetylene (PA) [11], and polycarbazol (PC) [12]. Unlike inorganic materials, redox polymers show properties such as low viscosity, low thermal conductivity, easy processing in versatile shapes, and adjustable molecular structures [13]. Their diversity in shapes and structures could also be beneficial for the conversion of polymeric compounds into widely used carbon species, i.e., carbon fibers, activated carbon, and graphene, etc. [14]. These materials confer great benefits, such as mechanical improvements or increased conductivity [15]. Furthermore, they can be derived from natural sources such as wood [16], which is mainly composed of cellulose, hemicellulose, and lignin [17].

Lignin is a highly complex aromatic biopolymer, which is usually found in larger quantities around the world, typically used as a source of fuel or an additive [18,19] in bio-mass material applications. Implementation of lignin within applications with higher added value has been extensively studied for decades, however, the field of energy storage systems can be added as a novel application, already holding numerous investigations in various topics, though, lignin as bulk so far only have been used for generating heat energy [20]. Due to its insulating nature, improvement in its electrochemical properties/redox activity could be challenging, nonetheless, lignin offers enriched vital functional groups like hydroxyl (–OH), methoxy (OCH_3_), aldehyde (CHO), and carbonyl (C=O) that support easy processing in synthetic monomers and polymers. The highly rich aromatic structure of lignin allows fabrication of low-cost, activated, and well-ordered carbons in distinctive shapes and forms [21,22,23,24]. Lignin has already been exploited in different battery systems as binder [25,26], electrolyte [27], and as an additive [28,29].

It has been established that modified and treated lignin could affect positively towards discharge performance of the battery [30,31]. In the meantime, the study of the impacts of different lignin components and the charge storage capacity of lignin have shown the redox activity (combined faradaic/non-faradaic charge storage) proving that by adding non-modified lignin, the capacity of the mixture could increase due to electrical double layer (EDL) charge storage that is usually dependent of the surface area. Hence, the final composite product can provide charge storage capacity depending on the mixing ratio and surface area. However, the highest capacity could be achieved via exposure of lignin functionalities towards electrolyte, homogeneity, and high surface area, even with the unmodified commercial one [32,33,34,35,36,37,38,39]. In order to allow faster transfer of charge storage, lignin needs suitable alterations, whether it is by chemical or physical inter-cross linking, thereby enhancing electronic conductivity and helping with the electroactive redox activity [40,41].

The main goal of this study is to achieve low cost and easy-processed lignin-based composite membranes with improved redox activity. The commercial unmodified organosolv lignin has been used, due to its higher relative amount of phenolic hydroxyl functional groups, although, freshly extracted kraft lignin has also been tested to observe the effect of sulfur groups over redox chemical reaction. Trials of different ratios and optimizations have been carried out, and the mixture was further stabilized with the help of non-ionic plasticizer polymers such as polyethylene oxide (PEO), which also help in providing extra –OH groups within the mixture. Mild reaction conditions and simple mixing techniques have been employed to prepare the blends of lignin to emit multiple modification reaction steps in favor of an affordable and ecological approach. A strong acidic nature polymer, Nafion^®^ has been considered for the easy cleavage of covalent bonds to enhance the possibility of inter-cross linkage that provides the adequate charge for the ionic transfer via chemical/physical interaction of lignin and, assuming that within the process of constant stripping/plating process, these membranes display the promising longer stripping/plating cycle life. To our knowledge, this is the first attempt to prepare lignin composite polymeric matrix with PEO adding a strong ionic acid, i.e., Nafion^®^ with mild conditions, the aim is to coerce potential lignin electrochemical redox activity by activating its quinone functionality. There have been several studies about the activation of methoxy group resulting quinone species via oxidation of guaiacyl (G) and syringyl (S) aromatic alcohol units [42,43,44]. Upon forming the quinone species, charge transfer process initiates within the surface of electrode and electrolyte. On that basis, we have considered the quinone as a major functionality of lignin interacting during the electrochemistry of the composite membranes when in electrolytic solutions under certain conditions. Nevertheless, Nafion^®^itself provides enough charge for the ionic transfer via chemical or physical interaction with lignin. The lignin composites were characterized via physico-chemical and electrochemical measurements to analyze the synergic arrangement of the composite.

## 2. Materials and Methods

Organosolv commercial lignin was purchased from Chemical Point UG (Oberhaching, Deisenhofen, Germany) and Nafion^®^ Perflourinated resin powder from Ion Power Inc. (The Chemours Company, München, Germany). Polyethylene oxide (PEO: M.W. 100,000), Dimethyl sulfoxide (DMSO), Ethanol (EtOH), and Sulfuric acid (H_2_SO_4_) were purchased from Sigma-Aldrich. Kraft lignin was freshly extracted from black liquor received by a local paper industry (Papelera Guipuzcoana de Zicuñaga, Hernani, Spain) of eucalyptus source.

### 2.1. Lignin

**Figure 1:**
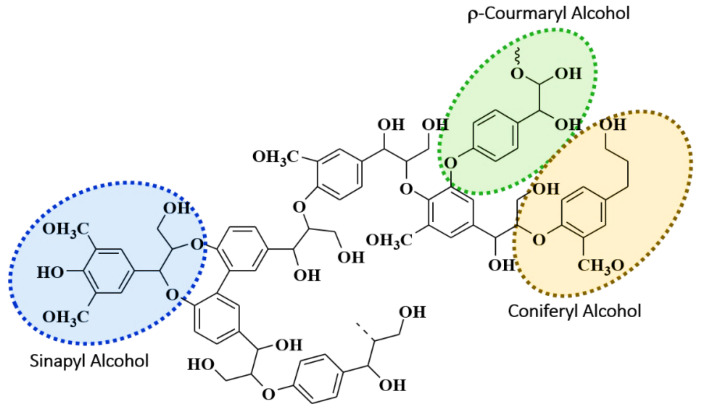
Chemical structure representation of lignin showing its major aromatic alcohol units.

Two types of lignins have been used in experimentation, commercially available organosolv lignin (Softwood) and freshly extracted kraft lignin from black liquor of local paper pulp industry (Hardwood). The general structure and major units of lignin have been shown in Figure 1. Lignin have three major composing aromatic alcohol units, sinapyl, coniferyl, and p-coumaryl (Figure 1), the difference between these alcohols is linkage of methoxy group on the phenol structure. These alcohol later form the monolignols of lignin, i.e., syringyl (S), guaiacyl (G), and p -hydroxyphenyl (H) [45,46,47,48,49].

The lignins have been thoroughly characterized within our research group “BioRP”, and the ratios and values of H, G, S, and ratio of S/G (Table 1) have been calculated via pyrolysis of the mentioned lignins.

### 2.2. Preparation of Lignin Based Composite Membranes

**Figure 2:**
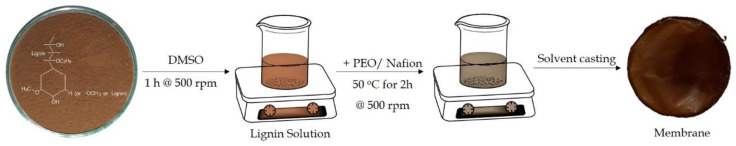
Illustration of the preparation process of Lignin based composite membranes.

The composite membranes were prepared by using DMSO solvent technique. Oven dried lignin was dissolved in different quantities in a closed vial (Table S1, Figure 2). PEO and Nafion^®^ polymer solution was prepared in acetonitrile under vigorous stirring, until the powder was completely dissolved. The mixture solution was transferred into the lignin solution, and the mixture was left at the reaction temperature of 50 °C with continuous stirring for 2 h. The concentration of mixture solution was maintained at 5 wt. %. OLPO, OLNF, and OL/KLPONF composite membranes were obtained by solvent casting the homogeneous lignin–polymer mixture in Teflon dish and dried at 40 °C.

The obtained dried membranes, light brown to darker brown in color, were punched out into 20 mm discs to be used as working electrodes in electrochemical characterizations. The mixture containing a higher percentage of lignin resulted in a thick gel to hard rock pieces, which were difficult to manage and analyze.

### 2.3. Characterization

The water uptake (WU), swelling ratio (SR), and gel content percentage (GC) test were carried out with oven dried composite membranes (~2.49 × 1.5 × 0.075) in de-ionized (DI) water at room temperature. After 24 h, the membranes were collected and measured to calculate the values of WU, SR, and GC percentage, equation S1, S2, and S3 have been used, respectively (Detailed procedure in supplementary information).

Fourier transform infrared spectra (FTIR) spectroscopy measurements were attained on a PerkinElmer Spectrum Two FT-IR Spectrometer equipped with Universal Attenuated Total Reflectance (UATR). All measurements were done ranging from 400 to 4000 cm^−1^ at room temperature with 64 average scans. Differential scanning calorimetry (DSC) measurements were carried out on DSC822e (Mettler Toledo) in the range of room temperature to 500 °C under N_2_ atmosphere with the heating rate of 10 °C min^−1^.

Electrochemical measurements were carried out at room temperature with mild flow of N_2_ gas using Solartron Multipotentiostat 1480 and Solartron mobrey SI 1260 (Impedance-Gain phase analyzer) in a conventional three-electrode electrochemical cell with a wide platinum foil as a counter electrode and an Ag/AgCl reference electrode. The electrochemical activity of the composite materials was evaluated in aqueous solutions of 1 M H_2_SO_4_. CV measurements were performed at different scan rate of 5, 10, 20, 30, and 50 mVs^−1^ in the range of −1 V to 1 V. EIS spectra were scanned within a frequency range of 0.01 Hz to 100,000 Hz with AC amplitude of 10 mV at room temperature.

Error of the repeated data of WU, SR, GC, and EIS have been calculated by statistical calculation as a function of standard deviations (SD) and ratio of variances to ensure the behavioral changes and variability within different composites.

## 3. Results

### 3.1. Water Uptake (WU), Swelling Ratio (SR) and Gel Content (GC) % Tests

**Figure 3:**
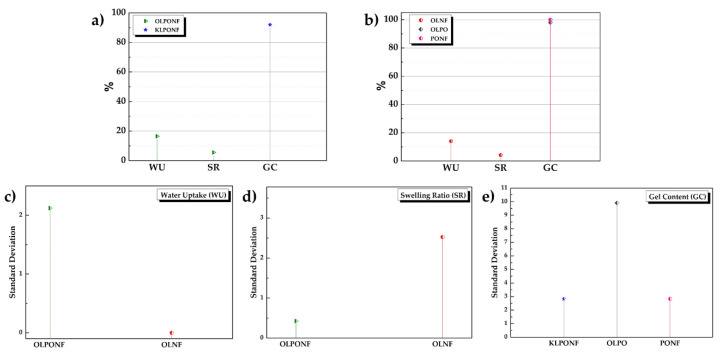
(**a**,**b**) Representation of the percentage rate of water uptake (WU), swelling ratio (SR), and gel content (GC) contents in different composite membranes. (**c**–**e**) Error statistical analysis via standard deviations (SD) with respect to their mean values.

The membranes were analyzed by water uptake and swelling ratio test, due to the presence of polymers in the lignin composite with relatively high hygroscopic ratio. Figure 3 shows the average WU, SR, and GC% of different membranes.

The water uptake capacity of lignin/polymer composite membranes showed lower rehydration ratios, probably due to the pore generation/swelling owing to their lower thickness. The thickness can be a crucial factor for the pore sizes and porosity of material. PONF, OLPO, and KLPONF exhibited gel content of 100, 98, and 92%, probably due to the sulfur group in kraft lignin and hygroscopic nature of the PEO. However, it might lead to poor mechanical properties and high-water ions permeability. Though, addition of OL lignin in PONF mixture increases the integrity of the membrane, perhaps owing to the successful crosslinking, providing an advantage of stronger mechanical properties. The error calculated via statistical analysis of the repeated samples during WU and SR tests shows that the behavior of each membrane is quite different depending on the composite mixture that plays an important role in their structural integrity, for example, in the case of WU and SR, OLPONF, and OLNF shows the difference of ~2–2.5 points, respectively. That could accord with the hygroscopic nature of PEO in OLPONF membrane to help absorb higher amount of water than OLNF. However, the physico-chemical intercross linkage between PEO and lignin in the presence of Nafion^®^ provides a firm wholeness to avoid membrane dissolution, which could be confirmed by the OLPO membrane’s total disintegration. Comparing the lignins, in the case of KLPONF, the membrane totally disintegrated, and thus the highest GC% and SD have been calculated, and as mentioned, structural integrity achieved via composite mixtures is totally dependant on their nature, which give a clue that this might be due to correlation of sulfur group in KL with sulphonic groups (–SO_3_H) of Nafion^®^. Thorough studies are needed to understand this phenomena.

### 3.2. Fourier Transform Infrared (FTIR) Spectroscopy and Differential Scanning Calorimetry (DSC)

**Figure 4:**
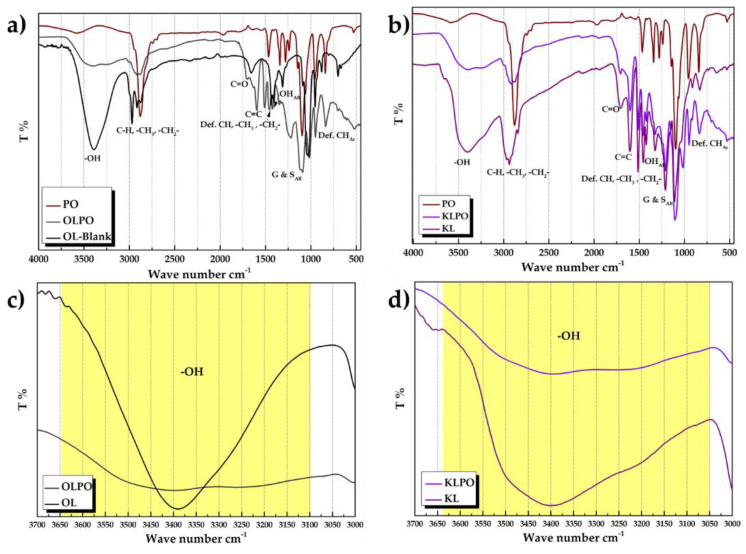
FTIR (Fourier transform infrared) spectra (**a**) Organosolv lignin-PEO (polyethylene oxide) (OLPO) composite in comparison to PEO (PO) and blank organosolv lignin (OL), (**b**) comparison of OLPO and OL to demonstrate shift in peak in the range of 3000–4000 cm^−1^, (**c**) Kraft lignin-PEO (KLPO) composite in comparison to PEO (PO) and blank kraft lignin (KL), (**d**) comparison of KLPO and KL to demonstrate shift in peak in the range of 3000–4000 cm^−1^.

FTIR spectra validates the incorporation of polymer components with lignin functionality and confirms the physico-chemical crosslinking. Figure 4 displays FTIR spectra of the pure lignin (OL/KL), PEO (PO), and lignin-PEO mixture (KL/OLPO, 80:20). The OLPO and KLPO mixture shows comparable peaks as of non-modified lignin (OL/KL) with a slight shift in the wavenumber. Lignin usually shows OH stretching vibration in the shape of a broad peak between 3045 and 3562 cm^−1^, basically due to the presence of alcoholic and phenolic hydroxyl groups, whereas the characteristic peaks of PEO appear around 3585 cm^−1^ and 1096 cm^−1^. In the composite mixture, a broad peak with slight shoulder corresponding to OH stretching appears between ~3673–3057 (OLPO) and 3714–3031 cm^−1^ (KLPO) [50]. Alkyl group (–CH, –CH_3_, –CH_2_–) has an intense band at ~2939 cm^−1^ and at 2844 cm^−1^, and aromatic C=C stretching at ~1599 and 1504 cm^−1^. A new band at 1594 cm^−1^ appeared, i.e., possibly corresponding to the stretching of aromatic ring of lignin, peaks of –OH bending vibrations of the aromatic ring appears at 1459, 1424, 1351, and 1328 cm^−1^ and C–O–C stretching vibration at 1210, 1108, and 1031 cm^−1^. In some literature, the particular peaks at 1328, 1210, and 1108 cm^−1^ were assigned to the vibrations of syringyl rings and guaiacyl rings. The band at 834 cm^−1^ in KL represents the deformation vibrations of C–H bonds in the aromatic rings, which usually corresponds to the aromatic –OH stretching, whereas aliphatic –CH stretching appears at ~2879 cm^−1^, –CH bending at 1466 and 1341 cm^−1^. C–O–H stretching shows a peak at 1279 cm^−1^ [51].

The shoulder of the broad peak at 3226 cm^−1^ could be due to the intermolecular –H bonds of aliphatic hydroxyl groups. The peak at 3404 cm^−1^ corresponds to intermolecular dimer OH peak. Intermolecular bonded OH peak at 3236 cm^−1^ is a shoulder appearing in OLPO and KLPO, even though KL shows the slight shoulder itself, however, in the case of the mixture, the low intensity of the band could prove the formation of intermolecular hydrogen bonding, confirming cross-linkage of lignin with PEO [52].

**Figure 5:**
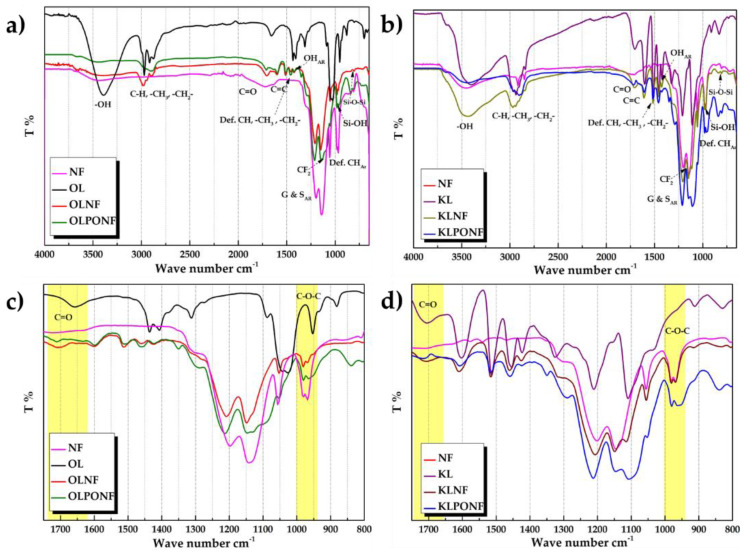
FTIR spectra of (**a**) OLPONF and OLNF composite in comparison to NF and OL, (**b**) comparison of C–O–R and C=O groups to demonstrate shift in peak. (**c**) KLPONF and KLNF composite in comparison to NF and blank kraft lignin, (**d**) comparison of C–O–R and C=O groups to demonstrate shift in peak.

FTIR spectra (Figure 5) of OLPONF and KLPONF has been compared with the Nafion^®^, OL, and KL spectra, and mostly the peak resembles the mixture of lignin and Nafion^®^, however, the peaks of PEO seems to be overlapped by the OLNF mixture. In the spectra, the peaks which seem to have a shoulder in the region of ~980.64 cm^-1^ in OL/KLNF and OL/KLPONF are probably corresponding to the C–O–C group of Nafion^®^ that usually appears slightly shifted (981.21 cm^−1^) in pure Nafion^®^. The peak at ~960 cm^−1^ corresponds to Si–OH, Si–O–Si at 804 cm^−1^ instead of 809 cm^−1^, and CF_2_ within the region of 1100–1200 cm^−1^. C=O peak has shifted from 1656 to 1712 cm^−1^, which in OL/KLNF also appears around same shift. The changing behavior and impact of Nafion^®^ on the functionality of lignin is still yet to be discovered in depth, however, the possible interconnection could be proven on the bases of slight shifting of C–O–C and C=O stretching vibration bands (Table S2) [53].

**Figure 6:**
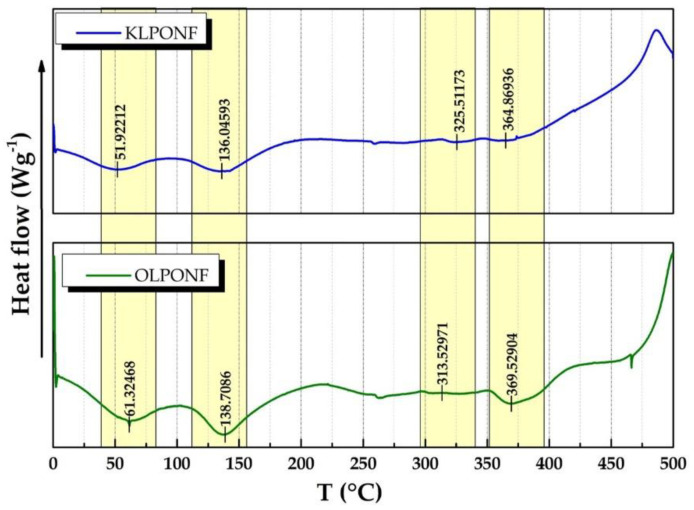
Differential scanning calorimetry (DSC) curves of lignin based composite membranes.

Differential scanning calorimetry (DSC) measurements of the lignin based composite membranes was performed to study the thermal changes within composites blends (Figure 6), which shows the characteristic peaks of PEO, lignin, and Nafion^®^. The endothermic curve following by exothermic peaks within the regions of 35–150 °C and 300–400 °C, usually, corresponds to fusion or melting and crystallization, respectively. However, KLPONF shows intense peaks, possibly due to the sulfur groups of kraft lignin or sulfonic acid (–SO_3_H) groups of Nafion^®^. These results will need further confirmation by thermogravimetric analysis (TGA) studies.

### 3.3. Conductivity Measurements

**Figure 7:**
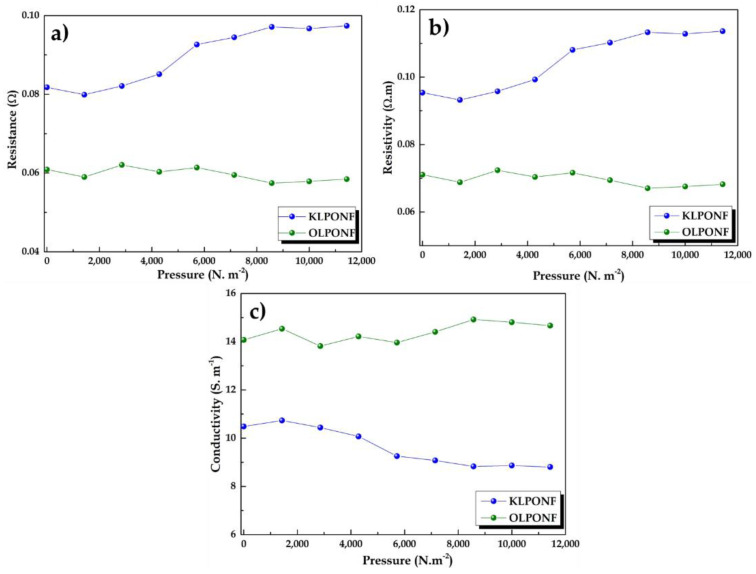
The variations of KLPONF and OLPONF membranes in terms of (**a**) resistance, (**b**) resistivity, and (**c**) conductivity with reference to applied pressure.

The pressure dependent resistance and conductivity plot as a function of resistivity of OLPONF and KLPONF are shown in Figure 7. The resistance of the KLPONF membrane seems to considerably alter depending on the pressure applied, however, the OLPONF membrane shows a more or less constant plateau, which could refer to its better mechanical properties as demonstrated in the wetting test. The conductivity of these membranes has been calculated within the range of 10–14 S m^−1^, which could be sufficient considering the fact that lignin is well-thought-out as an insulating material. The conductivity measurements are yet to be confirmed by the help of EIS techniques.

**Figure 8:**
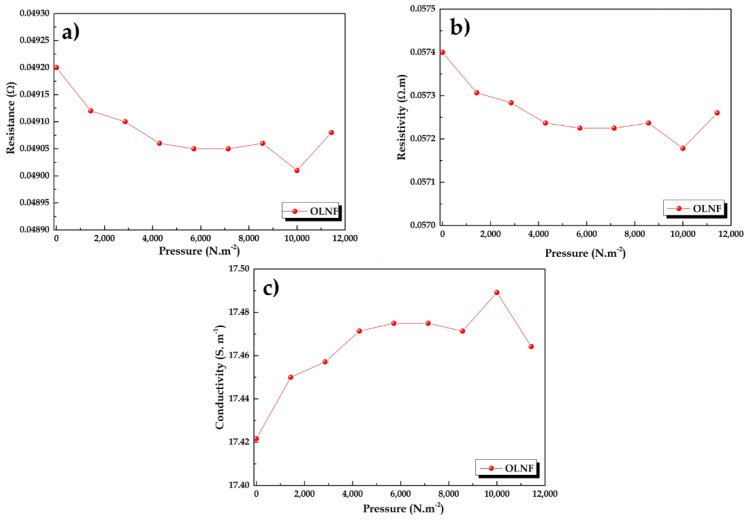
The variations of OLNF membranes in terms of (**a**) resistance, (**b**) resistivity, and (**c**) conductivity with reference to applied pressure.

In order to test the theory of increased conductivity of lignin via composite mixture within an ionically rich polymer matrix, the tests have been repeated with the membrane only with lignin and Nafion^®^ (OLNF) (Figure 8). The attempts to record the resistance and conductivity of the KLNF mixture membrane was unsuccessful due to the brittleness of the membrane, which broke upon applying pressure, however, sometimes we achieved a gel type membrane that usually stuck to the surface of Cu electrodes and made it quite difficult to follow the calculation procedure.

The OLNF membrane shows more improved conductivity than OLPONF, i.e., ~17–18 S m^−1^. Although, this humble change in conductivity opens up the vast door for the diverse possibilities to caper and tune it as required.

An attempt to measure the conductivity in a liquid phase (organic solvent) was done, in order to have an assurance of the direct influence of Nafion^®^ on the conductivity (Table 2). It seems to decrease up to ~10^−4^, owing to the fact that Nafion^®^’s hydrophilic sulfonate groups could improve the solubility of quinone in water resulting ionization and production of aromatic anion. However, in the case of organic solvents, the ionization of quinone was restricted, which affected the conductivity. Usually, in the case of liquids, the conductivity shown within a mixture solution is proportional to its ion concentration.

To explain the loss of hydrophilic groups during the solution submersion, the conductivity has been noted after the wetting test, the OLPONF membranes were chosen on the basis of their higher mechanical intactness, the resistance and resistivity surprisingly increased after submersion, and there was a drop in conductivity that remain constant after applying pressure within the range of 1000–12,000 Nm^−2^ (Figure S2).

### 3.4. Cyclic Voltammetry (CV) Measurements

**Scheme 1:**
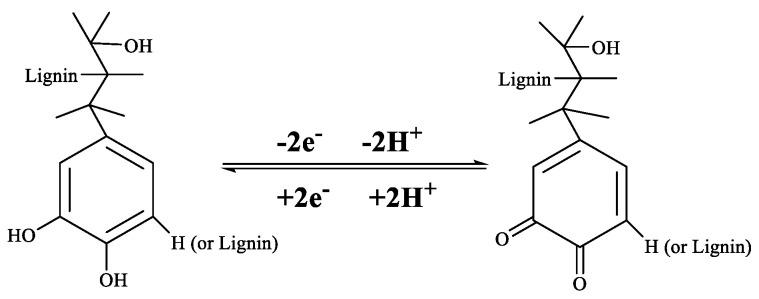
Redox activity of quinone/hydroquinone functionalities during the charge–discharge process.

**Figure 9:**
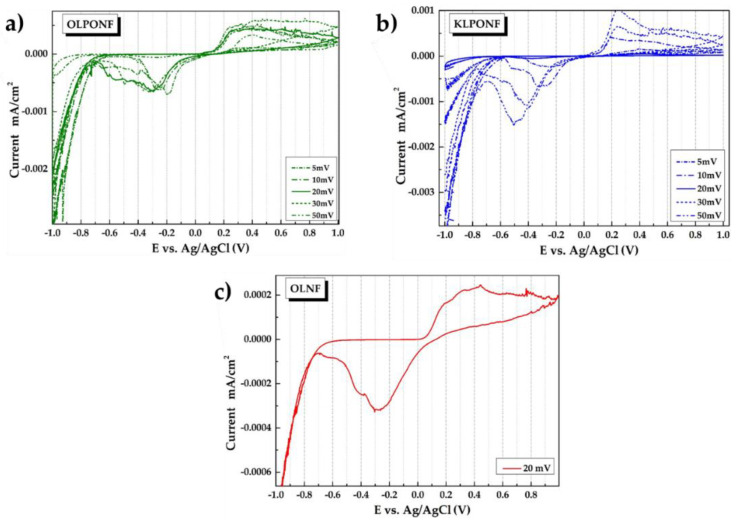
Shows the four cyclic voltammetry (CV) profiles of the OLPO, OLNF, OLPONF, and KLPONF electrodes performed in the window of −1 V to 1 V potential range at the scan rate of 20 mVs^−1^.

Figure 9 shows the cyclic voltammetry (CV) profiles performed in the window of −1 to 1 V potential range for the OLPONF and KLPONF as WE at different scanning rates to follow the redox activity corresponding to the quinone functionality of lignin (OL/KL) composite mixture. Meanwhile, the profiles for OLNF electrodes were measured at the scan rate of 20 mVs^−1^ for the comparison. Usually, the quinone moiety shows a sharp oxidation peak at ∼0.6 V and an obvious reduction peak at ∼0.4 V, however, the oxidation and reduction peak appears to shift to ∼0.37–4 V and ∼0.3–0.6 V vs. Ag/AgCl (Sat. KCl), respectively, possibly demonstrating the coordination interactions of ether oxygen of PEO and Nafion^®^’s carbonyl oxygen atoms of lignin, it might also be the hindrance due to SO_3_H (sulfonic acid) group of Nafion^®^. The reversible faradic quinone/hydroquinone conversion reaction has been demonstrated in Scheme 1, where quinone functionality lose and gain 2 electrons/protons during the discharge and charge process.

The sweep of different potential rates has been performed from 5–100 mVs^−1^, however, there wasn’t any significant changes in the current rate, which could be due to the possibility of constant ion-adsorption dependent redox processes. This constant behavior could be explained by the fact that the acidic electrolyte has been changed after each experiment, which could provide the constant ionic mobility. The constant current profile could also be due to a constant ratio of lignin in the sample electrodes.

The voltammogram of the samples with higher concentrations, i.e., thicker gel like lignin composite shows very little redox activity, probably due to the insulating nature of lignin and the disintegration within the electrolytic solution, however, the low amount of ionic polymer wouldn’t have any significant contribution to the electron storage.

### 3.5. Electrochemical Impedance Spectroscopy (EIS)

To further interpret the electrochemical performance, galvanostatic charging and discharging of the lignin composite mixtures was conducted. The active mass loading of lignin has been maintained at 4 mg/mL. The charge-discharge 10 cycle curves at the current density of 100 mA.cm^−2^ between −1 and 1 V display different shapes compared to traditional capacitive ion storage, which seems quite incomprehensible. The curves look almost symmetrical, indicating the charge storing ability of these composites. Excellent cycling stability of the electrode was obtained, which could be due to the unique structural features of the composites. Nafion^®^ could act as a barrier and keep the lignin intact within the electrode, which could avoid the loss of active mass in the electrolyte, and due to its ionic conductivity, it could facilitate the charge transfer process by easing the efficient electron transport pathway. Additionally, the porous PEO polymer could work as a buffer, which provides the strain relaxation and volume change, enabling the easy access of the electrolyte passage. Due to incomprehension and to be certain EIS measurements were conducted before and after the GCPL cycling measurements.

**Scheme 2:**
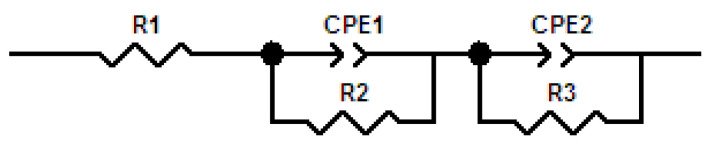
Series equivalent electric circuit model created on the basis of structural impedance behavior of lignin based composite membranes.

Experimental impedance results are represented using Nyquist curves, where a non-linear adjustment techniques CNLS (complex nonlinear least square) was applied to a series equivalent circuit model (Scheme 2). The ECM series shows different components, where R1 represents the resistance of the assembly formed by electrolyte, working electrode, and reference electrode. R2 is the interface resistance related to encapsulation of lignin within PEO and Nafion^®^ structure. In order to represent the hydrophobic and heterogeneous structure of lignin mixture, CPE1 (constant phase element) have been used. CPE elements are able to reproduce the inhomogeneity related to the porous structure of the electrode, mass transfer phenomena, or charge transfer reactions. The characteristics of the double layer are associated to the values of R3 (charge transfer resistance) and CPE2 (constant phase element) [54,55,56]. Two capacitive arcs have been obtained, whose characteristic time constants correspond to two CPE-R pairs. The high frequency time constant, associated with the pair R2/CPE1, reflects the characteristics of the pore resistance and the low-frequency constant, reflects the characteristics of the load transfer resistance, associated with the pair R3/CPE2. The experimental impedance values fit well with the series ECM.

**Figure 10:**
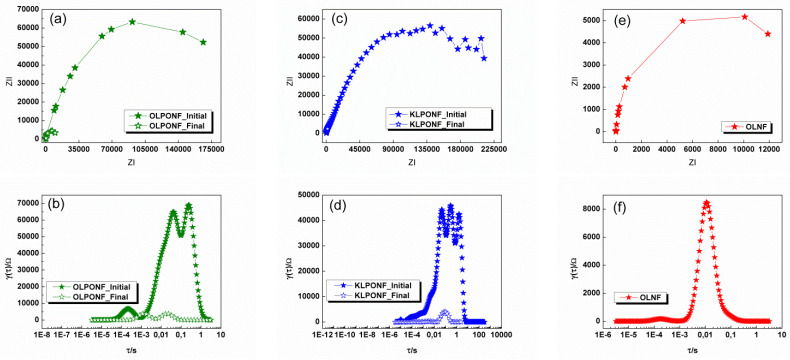
Nyquist and distribution of the relaxation times (DRT) plots corresponding to the impedance spectra of the; (**a**,**b**) OLPONF, (**c**,**d**) KLPONF, and (**e**,**f**) OLNF electrodes before and after the GCPL cycling measurements, respectively.

To understand the physico-chemical processes within different time constants in the EIS spectra (Figure 10), the distribution of the relaxation times (DRT) of the experimental impedance data was calculated [56,57,58,59,60]. Relaxation times obtained when fitting the impedance data to the ECM model, with respect to the times obtained when representing the same impedance data through the polarization processes, and (DRT). From the values of the different CPE1 and CPE2 the values of the capacitances can be obtained [61,62] as shown in Tables S2 and S5.

When comparing the time constants (τ) obtained through experimental and DRT calculations, it can be seen that the obtained results differ, the DRT values have been shown in Table S3. After cycling, KLPONF shows only a low frequency relaxation time, and the high frequency time constant is absent within the DRT representation. The behavior of all lignin samples follows a decreasing pattern in the evolution of impedance. In the OLPONF and KLPONF samples, this impedance drop is much more accentuated, greatly decreasing the interface resistance related to the encapsulation of lignin within the structure of PEO and Nafion^®^. The sample that best maintains its initial and final values and characteristics in a chemical environment is OLNF, possibly due to their structural integrity as shown by WU and SR% test results. To validate the EIS data, the statistical analysis of repeated samples have been calculated (Figure S3), which reflects that the compounds are highly electrochemically responsive depending on their composite mixture (surprisingly, the KLPONF and OLPONF composites have shown more or less similar behavior in SD as well as in variance, however, the OLNF have been proven to show the highest distributive values), which could be due to the behavioral changes of the biomass polymer (lignin) within the polymeric matrix, i.e., difference between their intercross linkages, however, these outcomes need much strict understandings.

### 3.6. Perspectives and Limitations

The main goal of this study is to develop lignin-based conductive composite materials and study their electrochemical properties for applications within electrochemical energy storage (EES) systems such as batteries, fuel cells, or supercapacitors. This study validates the idea of activation of lignin redox properties via simple modification in the polymeric matrix. However, lignin possesses diverse functionalities and its richness in aromaticity is yet to be explored, thus, our major goal is to provide a fully organic biomass conductive polymeric matrix via playing and tuning the functional groups of lignin using only simple and low-cost techniques that could open a door towards economical energy storage systems. Although this study proposes an easy and inexpensive casting method to prepare composite membranes, the thickness control is the major limitation of this methodology that could lead to the probability of error despite following the exact steps and procedure. Thus, further studies will also be focused on the membrane casting optimization to establish internal and external validity of the result of unanticipated challenges that could emerged during the study.

## 4. Conclusions

Stable and functional lignin-based composite (OL/KL-PONF) membranes were prepared by an easy mixing and solution casting method to be used as cheap electrodes. The present work shows consecutive characterization of the membranes to conclude the improved conductivity, the structural functionality, and integrity. A strong ionic acid in mild reaction conditions was added to facilitate the pathway of oxidation and activation of the quinone reversible (2e^−^/2H^+^) redox cycling. The FTIR spectroscopy confirms the formation of the composite mixture, where the shift in the vibration band of C–O–C and C=O stretching and appearance of Si–OH, Si–O–Si, and CF_2_ was observed due to incorporation of Nafion^®^. Whereas, electrochemical processes and CV scans confirm the redox couple behavior and stability over repetitive cycling of the composite membranes. Further studies will continue on the in-depth understanding of lignin functionalities behavior along with exploration of potential modification routes, and as Nafion^®^ is an expensive polymer, the alternatives are needed to be studied in order to advance in the optimization of the composite membranes as well as of the electrochemical process.

## Figures and Tables

**Table 1:** Source, origin, and monolignols ratios of used lignins.

Type	Source	Origin	Ratios			
			S	G	H	S/G
Organosolv	Commercial	Softwood	15.57	42.77	9.28	0.36
Kraft	Industry	Hardwood	64.88	32.20	1.42	2.01

**Table 2:** Composite membranes conductivity comparison in solid and liquid media.

Solid	Liquid
1.742 × 10^−1^ S.cm^−2^	1.79 × 10^−4^ S.cm^−2^

## Data Availability

Data is contained within the article or supplementary material.

## Supplementary Materials

The following are available online at https://www.mdpi.com/2073-4360/13/4/643/s1, Figure S1: (a) Assembly diagram of the ICR test unit. (b) Experimental setup used in the measurement for ICR tests. Table S1: Demonstration of different ratios of lignin based composite membranes. Table S2: Representation of the peak position of different functional groups of lignin and Nafion® in composite membranes with comparison to blank lignin. Figure S2: The comparison of OLPONF membrane before and after the wetting test experiments, shows (a) Resistance, (b) Resistivity and (c) Conductivity with reference to applied pressure. Figure S3: Statistical analysis via standard deviation (SD) and ratio of variances plots corresponding to the Z’ (real) and Z” (Imaginary)-axes of the EIS spectra of (a–b) OLPONF, (c–d) KLPONF and (e–f) OLNF electrodes before the GCPL cycling measurement. Table S3: Electrical parameters adjustment of the impedance spectra of OLPONF, and KLPONF, to the electric equivalent circuit before and after electrochemical cycling. Table S4: Electrical parameters adjustment of the impedance spectra of OLPONF, and KLPONF before and after electrochemical cycling. Table S5: (a) Electrical parameters adjustment and (b) DRT values adjusted to the experimental impedance spectra of OLNF before and after electrochemical cycling. Table S6: The specific capacitance calculated from experimental data.
