# Supplementary material for: Electrochemical Activity of Lignin Based Composite Membranes
Source: Polymers (Basel). 2021 Feb 21;13(4):643. doi: 10.3390/polym13040643 (PMC7926618; doi:10.3390/polym13040643)
Supplement: Supplementary file 1 [file polymers-13-00643-s001.pdf]

polymers MDPI

Article

Electrochemical Activity of Lignin Based Composite Membranes.

Marya Baloch [1], Mikel Alberro Astarbe [2] and Jalel Labidi [1,*]

1 Department of Chemical and Environmental Engineering, Faculty of Engineering, Gipuzkoa, Donostia-San Sebastian; marya.baloch@ehu.eus, jalel.labidi@ehu.eus
2 Department of Electronic Technology, Faculty of Engineering, University of the Basque Country (UPV/EHU), Plaza Europa 1, 20018, San Sebastian, Spain; mikel.alberro@ehu.eus
* Correspondence: jalel.labidi@ehu.eus; Tel.: +34-943-01-7178

Received: date; Accepted: date; Published: date

Graphical Abstract:

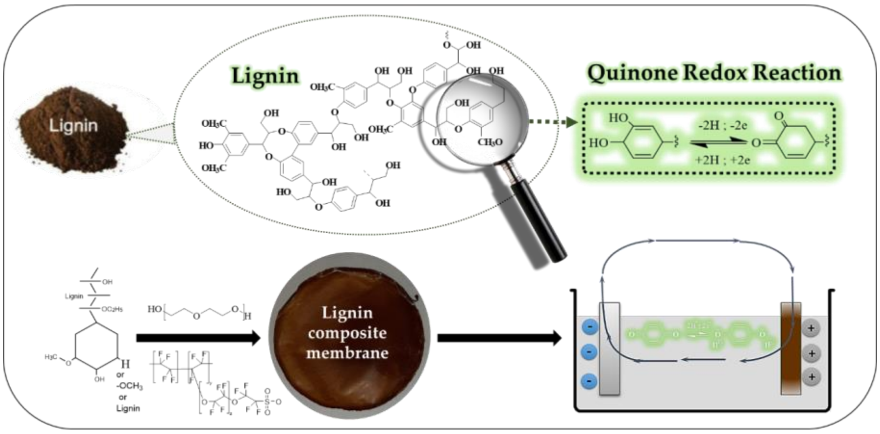

Key Highlights:

- Successful fabrication of lignin polymeric composite membrane electrodes via facile and low-cost method.
- Exploration of lignin's quinone functionality and its electrochemical activity.
- Achievement of enhanced functionality and improved conductivity of lignin by mixture composites.

Abstract: Our society's most pressing challenges like high CO_2 emission and the constant battle against energy poverty needs a clean and easier solution to store and utilize the renewable energy resources. However, recent electrochemical components are expensive and harmful for the environment, which restricts their widespread deployment. This study proposes an easy method to synthesize and fabricate composite membranes with abundantly found biomass lignin polymer to replace conventional costly and toxic electrode materials. Easier manipulation of lignin within the polymeric matrix could provide the improved composite to enhance its electrochemical activity. Our major focus is to activate, the quinone moiety via oxidation in the polymeric mixture using a strong ionic acid. The physico-chemical and electrochemical characterizations of two different lignins with

different polymeric mixture compositions have been carried out to confirms that the redox properties of pure unmodified lignin could be achieved via intrinsic mutual sharing of the structural properties and intercross linkage leading to improved integrity and redox activity/conductivity.

Keywords: Batteries, Lignin, Inexpensive, Environmentally Friendly, Quinone, Redox Activity.

---

1. Introduction

Electric energy storage systems are widely demanded by various sectors both for their great versatility and advantages as environmentally sustainable alternatives such as storage of energy from renewable resources.[1,2] With growing population, the CO_2 emissions from internal combustion engines are increasing, with a terrible effect both on personal health and the environment. From electrochemical energy storage, batteries are the systems with the highest storage capacity, due to their high energy density.[3–5] Although, batteries are efficient, convenient, reliable and easy to use, their useful life and autonomy are limited, in addition to their doubtful sustainability in terms of materials, since the materials used (both metals and non- metals) can generate various polluting constituents during the process.[6] Therefore, the practicality of these inorganic materials is limited by various factors such as scarcity, high cost, toxicity and difficulty of processing procedures. It is essential to find a replacement of these materials with the materials that are abundant and sustainable i.e. organic polymers.

The redox polymers have gained popularity as a replacement to harmful battery components due to their non-toxic nature and enhanced electrical properties such as poly (3,4-ethylenedioxyphene) (PEDOT),[7] polyaniline (PANI),[8] polythiophene (PTh),[9] polypyrrol (PPy),[10] polyacetylene (PA),[11] polycarbazol (PC) [12].Unlike inorganic materials, they show properties such as low viscosity, low thermal conductivity, easy processing in versatile shapes, and adjustable molecular structures.[13] However, conversion of polymeric compounds into carbon is widely used, due to their diversity in shapes and structures i.e. carbon fibres, activated carbon, and graphene, etc.[14] These materials confer great benefits, such as mechanical improvements or increased conductivity.[15] Furthermore, they can be derived from natural sources such as wood,[16] which is mainly composed of cellulose, hemicellulose and lignin. [17]

Lignin is a highly complex aromatic biopolymer, which is usually found in larger quantities around the world, typically used as a source of fuel or an additive[18,19] in bio-mass material applications. Implementation of lignin within applications with higher added value has been extensively studied for decades, however, the field of energy storage systems can be added as a novel application already holding numerous investigations in various topics, though, lignin as bulk so far only have been used for generating heat energy.[20] Due to its insulating nature, improvement in its electrochemical properties/ redox activity could be challenging, nonetheless, lignin offers an enriched vital functional groups like hydroxyl (-OH), methoxy (OCH_3), aldehyde (CHO) and carbonyl (C=O) that supports easy processing in synthetic monomers and polymers. The highly rich aromatic structure of lignin allows fabrication of low-cost, activated and well-ordered carbons in distinctive shapes and forms.[21–24] Lignin has already been exploited in different battery systems as binder,[25,26] electrolyte[27] and as an additive,[28,29].

It has been established that modified and treated lignin could affect positively towards discharge performance of the battery.[30,31] In the meantime, the study of the impacts of different lignin components and the charge storage capacity of lignin have shown the redox activity (combined faradaic/non-faradaic charge storage)proving that by adding non-modified lignin, the capacity of the

mixture could increase due to electrical double layer (EDL) charge storage that is usually dependent of the surface area. Hence, the final composite product can provide charge storage capacity depending on the mixing ratio and surface area. However, the highest capacity could be achieved via exposure of lignin functionalities towards electrolyte, homogeneity and high surface area, even though with the unmodified commercial one.[32–39] In order to allow faster transfer of charge storage, lignin needs suitable alterations, whether it is by chemical or physical inter-cross linking, thereby, enhancing electronic conductivity and helping with the electroactive redox activity[40,41].

The main goal of this study is to achieve low cost and easy-processed lignin based composite membranes with improved redox activity. The commercial unmodified organosolv lignin has been used, due to its higher relative amount of phenolic hydroxyl functional groups, although, freshly extracted kraft lignin has also been used to observe the effect of sulphur groups over redox chemical reaction of. Trials of different ratios and optimization has been carried out, the mixture was further stabilise with the help of non-ionic plasticizer polymer such as polyethylene oxide (PEO), which also help in providing extra –OH groups within the mixture. Mild reaction conditions and simple mixing techniques have been employed to prepare the blends of lignin to emit multiple modification reaction steps in favour of anaffordable and ecological approach. A strong acidic nature polymer, Nafion® has been considered for the easy cleavage of covalent bonds to enhance the possibility of inter-cross linkage that provides the adequate charge for the ionic transfer via chemical/ physical interaction of lignin and assuming that within the process of constant stripping/plating process, these membranes displays the promising longer stripping/plating cycle life. To our knowledge, this is the first attempt to prepare lignin composite polymeric matrix with PEO adding a strong ionic acid i.e. Nafion® with mild conditions, the aim is to coerce potential lignin electrochemical redox activity by activating its quinone functionality. There have been several studies about the activation of methoxy group resulting quinone species via oxidation of guaiacyl (G) and syringyl (S) aromatic alcohol units.[42–44] Upon forming the quinone species, charge transfer process initiates within the surface of electrode and electrolyte. On that basis, we have considered the quinone as a major functionality of lignin interacting during the electrochemistry of the composite membranes when in electrolytic solutions under certain conditions. Nevertheless, NF, itself provide enough charge for the ionic transfer via chemical or physical interaction with lignin. The lignin composites were characterisedcharacterized via physico-chemical and electrochemical measurements to analyseanalyze the synergic arrangement of the composite.

Formatted: Font: Not Bold

Formatted: Font: Not Bold

Formatted: Font: Not Bold

2. Materials and Methods

Organosolv commercial lignin was purchased from Chemical Point UG (Oberhaching, Germany) and Nafion® Perflourinated resin powder from Ion Power Inc. (The Chemours Company). Polyethylene oxide (PEO: M.W. 100,000), Dimethyl sulfoxide (DMSO), Ethanol (EtOH), and Sulfuric acid (H_2SO_4) were purchased from Sigma-Aldrich. Kraft lignin was freshly extracted from black liquor received by a local paper industry (Papelera Guipuzcoana de Zicuñaga) of eucalyptus source.

2.1. Lignin:

Two types of lignins have been used in experimentation, commercially available organosolv lignin (Softwood) and freshly extracted kraft lignin from black liquor of local paper pulp industry (Hardwood). The general structure and major units of lignin have been shown in figure 1. Lignin have three major composing aromatic alcohol units, sinapyl, coniferyl, and p-coumaryl (Figure 1), the difference between these alcohols is linkage of methoxy group on the phenol structure. These alcohol later form the monolignols of lignin i.e. syringyl (S), guaiacyl (G) and p -hydroxyphenyl (H). [45–49]

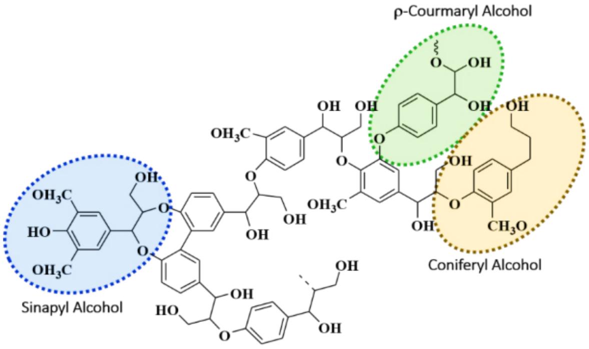

Figure.1: Chemical structure representation of lignin showing its major aromatic alcohol units.

The lignins have been thoroughly characterized within our research group "BioRP", the ratios and values of H, G, S, and ratio of S/G (Table. 1) have been calculated via pyrolysis of mentioned lignins.

Type	Source	Origin	Ratios			
			S	G	H	S/G
Organosolv	Commercial	Softwood	15.57	42.77	9.28	0.36
Kraft	Liquor paper industry	Hardwood	64.88	32.20	1.42	2.01

Table 1: Source, Origin and monolignols ratios of used lignins.

2.2. Preparation of Lignin based composite membranes

The composite membranes were prepared by using DMSO solvent technique. Oven dried lignin was dissolved in different quantities in a closed vial (Table S1, Figure 2). PEO and Nafion® polymer solution was prepared in acetonitrile under the vigorous stirring, until the powder was completely dissolved. The mixture solution was transferred into the lignin solution, the mixture was left at the reaction temperature of 50 °C with continuous stirring for 2 h. The concentration of mixture solution was maintained at 5 wt. %. OLPO, OLNF, and OL/KLPONF composite membranes were obtained by solvent casting the homogeneous lignin-polymer mixture in Teflon dish and dried at 40 °C.

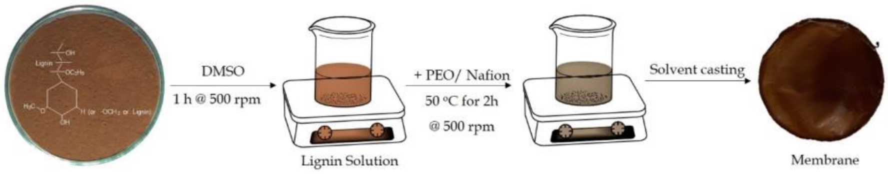

Figure.2: Illustration of the preparation process of Lignin based composite membranes.

The obtained dried membranes, light brown to darker brown in colour were punched out into 20mm discs to be used as working electrodes in electrochemical characterizations. The mixture containing higher percentage of lignin resulted into a thick gel to hard rock pieces, which were difficult to manage and analyze.

2.3. Characterization

The water uptake (WU), swelling ratio (SR) and gel content percentage (GC) test were carried out with oven dried composite membranes (~2.49 x 1.5 x 0.075) in de-ionised (DI) water at room temperature. After 24 hours the membranes were collected and measured to calculate the values of WU, SR and GC percentage equation S1, S2 and S3 have been used, respectively (Detailed procedure in supplementary information).

Fourier transform Infrared spectra (FTIR) Spectroscopy measurements were attained on a PerkinElmer Spectrum Two FT-IR Spectrometer equipped with Universal Attenuated Total Reflectance (UATR). All measurements were done ranging from 400 to 4000 cm^{-1} at room temperature with 64 average scans. Differential scanning calorimetry (DSC) measurements were carried out on DSC822e (Mettler Toledo) in the range of room temperature to 500°C under N_2 atmosphere with the heating rate of 10°C min^{-1}.

Electrochemical measurements were carried out at room temperature with mild flow of N_2 gas using Solatron Multipotentiostat 1480 and Solatron mobrey SI 1260 (Impedance-Gain phase analyser) in a conventional three-electrode electrochemical cell with a wide platinum foil as a counter electrode and an Ag/AgCl reference electrode. The electrochemical activity of the composite materials was evaluated in aqueous solutions of 1 M H_2SO_4. CV measurements were performed at different scan rate of 5, 10, 20, 30, and 50 mVs^{-1} in the range of -1V to 1V. EIS spectra were scanned within a frequency range of 0.01Hz to 100000 Hz with AC amplitude of 10 mV at room temperature.

Error of the repeated data of WU, SR, GC and EIS have been calculated by statistical calculation as a function of standard deviations (SD) and ratio of variances to ensure the behavioural changes and variability within different composites.

3. Results

3.1. Water Uptake (WU), Swelling Ratio (SR) and Gel Content (GC) % tests

The membranes were analyzed by water uptake and swelling ratio test, due to presence of polymers in the lignin composite with relatively high hygroscopic ratio. The figure 3 shows the average WU, SR and GC % of different membranes.

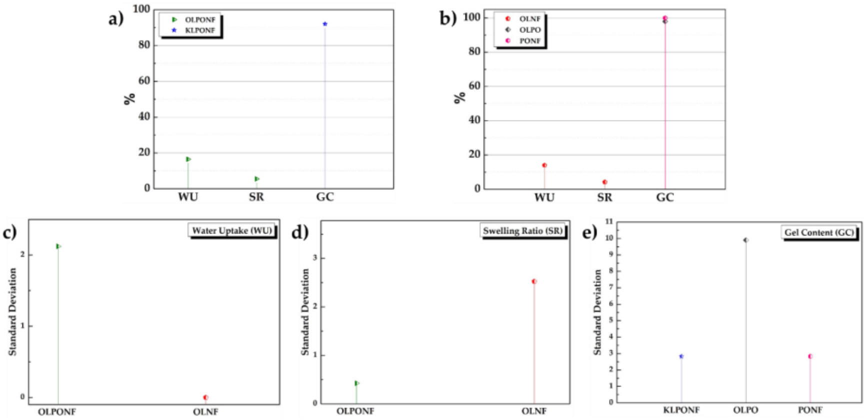

Figure 3: (a-b) Representation of the percentage rate of WU, SR and GC contents in different composite membranes, (c-e) Error statistical analysis via standard deviations (SD) with respect to their mean values.

The water uptake capacity of lignin/ polymer composite membranes showed lower rehydration ratios, probably due to the pore generation/swelling owing to their lower thickness. The thickness can be a crucial factor for the pore sizes and porosity of material. PONF, OLPO and KLPONF exhibited Gel content of 100, 98 and 92%, probably due to the sulphur group in kraft lignin and hygroscopic nature of the PEO. Though, it might lead to the poor mechanical properties and high-

water ions permeability. However, addition of OL lignin in PONF mixture increases the integrity of the membrane, perhaps owing to the successful crosslinking, providing an advantage of stronger mechanical properties. The error calculated via statistical analysis of the repeated samples during WU and SR tests shows that the behavior of each membrane is quite different depending on the composite mixture that plays an important role in their structural integrity, for example, in the case of WU and SR, OLPONF and OLNF shows the difference of ~2-2.5 points, respectively. That could accord with the hygroscopic nature of PEO in OLPONF membrane to help absorb higher amount of water than OLNF. However, the physico-chemical intercross linkage between PEO and lignin in the presence of NF provides a firm wholeness to avoid membrane dissolution, which could be confirmed by OLPO membrane's total disintegration. Comparing the lignins, in the case of KLPONF, the membrane totally disintegrated and thus the highest GC % and SD have been calculated, as mentioned, structural integrity is achieved via composite mixtures is totally depend on their nature, which give a clue that this might be due to correlation of sulphur group in KL with sulphonic groups (-SO_3H) of NF. The thorough studies are needed to understand this phenomena.

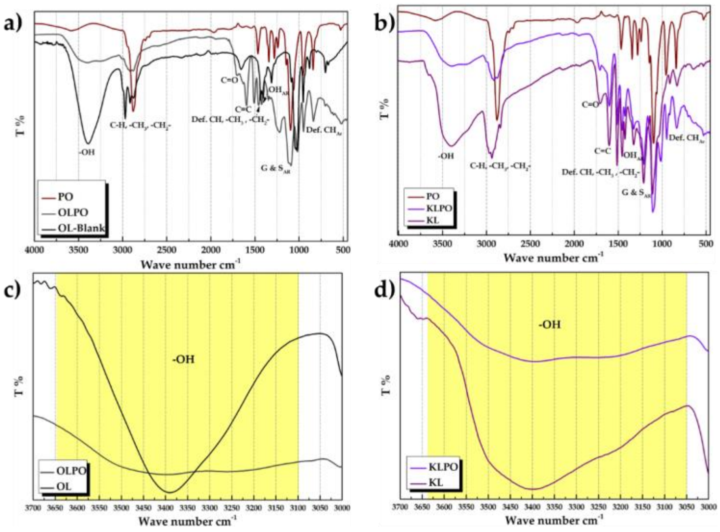

Figure 4: FTIR spectra a) Organosolv lignin-PEO (OLPO) composite in comparison to PEO (PO) and blank organosolv lignin (OL) b) comparison of OLPO and OL to demonstrate shift in peak in the range of 3000-4000cm^{-1} c) Kraft lignin-PEO (KLPO) composite in comparison to PEO (PO) and blank kraft lignin (KL) d) comparison of KLPO and KL to demonstrate shift in peak in the range of 3000-4000cm^{-1}.

3.2. Fourier Transform Infra-Red (FTIR) Spectroscopy and Differential Scanning Calorimetry (DSC)

FTIR spectra validates the incorporation of polymer components with lignin functionality and confirms the physico-chemical crosslinking. Figure. 4 displays FTIR spectra of the pure lignin (OL/KL), PEO (PO) and lignin-PEO mixture (KL/OLPO, 80:20) mixture. OLPO and KLPO mixture shows comparable peaks as of non-modified lignin (OL/KL) with slight shift in the wavenumber. Lignin usually shows OH stretching vibration in a shape of broaden peak between 3045 and 3562

cm^{-1}, basically due to the presence of alcoholic and phenolic hydroxyl groups, whereas the characteristic peaks of PEO appears around 3585 cm^{-1} and 1096 cm^{-1}. In the composite mixture, a broad peak with slight shoulder corresponding to OH stretching appears between ~3673-3057 (OLPO) and 3714-3031 cm^{-1} (KLPO).[50] Alkyl group (-CH, $-CH_3$, $-CH_2$-) has an intense band at ~ 2939 cm^{-1} and at 2844 cm^{-1}, and aromatic C=C stretching at ~1599 and 1504 cm^{-1}. A new band at 1594 cm^{-1} appeared i.e. possibly corresponding to the stretching of aromatic ring of lignin, peaks of -OH bending vibrations of the aromatic ring appears at 1459, 1424, 1351 and 1328 cm^{-1} and C-O-C stretching vibration at 1210, 1108 and 1031 cm^{-1}. In some literature, the particular peaks at 1328 cm^{-1}, 1210 cm^{-1}, and 1108 cm^{-1} were assigned to the vibrations of syringyl rings and guaiacyl rings. The band at 834 cm^{-1} in KL represents the deformation vibrations of C–H bonds in the aromatic rings., which usually corresponds to the aromatic -OH stretching, whereas aliphatic -CH stretching appears at ~ 2879 cm^{-1}, -CH bending at 1466 cm^{-1} and 1341 cm^{-1}. C-O-H stretching shows a peak at 1279 cm^{-1}.[51]

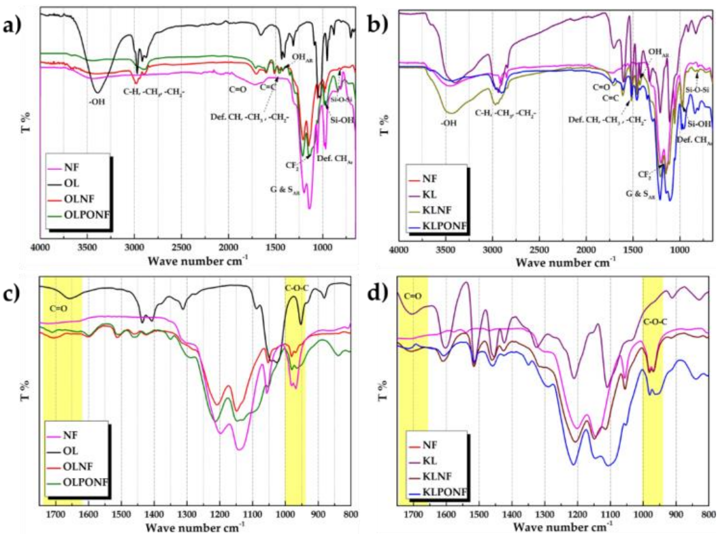

Figure 5: FTIR spectra a) OLPONF and OLNF composite in comparison to NF and OL b) comparison of C-O-R and C=O groups to demonstrate shift in peak. c) KLPONF and KLNF composite in comparison to NF and blank kraft lignin d) comparison of C-O-R and C=O groups to demonstrate shift in peak.

The shoulder of the broad peak at 3226 cm^{-1} could be due to the intermolecular –H bonds of aliphatic hydroxyl groups. The peak at 3404 cm^{-1} corresponds to intermolecular dimer OH peak. Intermolecular bonded OH peak at 3236 cm^{-1} is a shoulder appearing in OLPO and KLPO, even though KL shows the slight shoulder itself, however in the case of the mixture the low intensity of the band could prove the formation of Intermolecular hydrogen bonding confirming cross-linkage of lignin with PEO.[52]

FTIR spectra (Figure 5) of OLPONF and KLPONF has been compared with the NF, OL and KL spectra, mostly the peak resembles the mixture of lignin and Nafion®, however, the peaks of PEO seems to be overlapped by the OLNF mixture. In the spectra, the peaks which seems to have a

shoulder in the region of ~980.64 cm^{-1} in OL/KLNF and OL/KLPONF are probably corresponding to the C-O-C group of Nafion® that usually appears slightly shifted (981.21 cm^{-1}) in pure NF. The peak at ~960 cm^{-1} corresponds to Si-OH, Si-O-Si at 804cm^{-1} instead of 809 cm^{-1}, and CF_2 within the region of 1100-1200 cm^{-1}. C=O peak has shifted from 1656cm^{-1} to 1712 cm^{-1}, which in OL/KLNF also appears around same shift. The changing behaviour and impact of NF on the functionality of lignin is still yet to be discovered in depth, however, the possible interconnection could be proven on the bases of slight shifting of C-O-C and C=O stretching vibration bands (Table S2).[53]

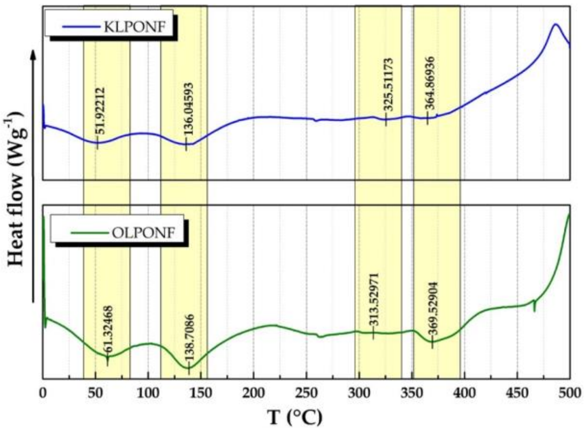

Figure. 6: Differential scanning calorimetry (DSC) curves of lignin based composite membranes.

Differential scanning calorimetry (DSC) measurements of the lignin based composite membranes was performed to study the thermal changes within composites blends (Figure 6), which shows the characteristic peaks of PEO, lignin, and Nafion ®. The endothermic curve following by exothermic peaks within the regions of 35-150°C and 300-400 °C, usually, corresponds to fusion or melting and crystallization, respectively. However, KLPONF shows intense peaks, possibly due to the sulphur groups of kraft lignin or sulfonic acid (-SO_3H) groups of Nafion®. These results will need further confirmation by thermogravimetric analysis (TGA) studies.

3.3. Conductivity measurements

The pressure dependent resistance and conductivity plot as a function of resistivity of OLPONF, and KLPONF are shown in figure 7a. The resistance of KLPONF membrane seems to considerably alter depending on the pressure applied, however, OLPONF membrane shows more or less constant plateau, which could be refer to its better mechanical properties as demonstrated in wetting test. The conductivity of these membranes has been calculated within the range of 10-14 S m^{-1}, which could be sufficient considering the fact that lignin is well-thought-out as an insulating material. The conductivity measurements are yet to be confirm by the help of EIS techniques.

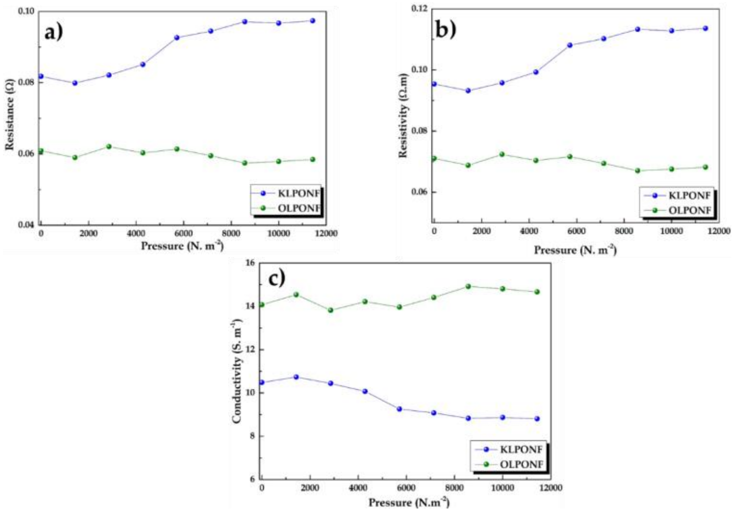

Figure. 7a: The variations of KLPONF and OLPONF membranes in terms of a) Resistance, b) Resistivity and c) Conductivity with reference to applied pressure.

In order to test the theory of increased conductivity of lignin via composite mixture within ionically rich polymer matrix, the tests have been repeated with the membrane only with lignin and nafion ® (OLNF) (Figure. 7b). The attempts to record the resistance and conductivity of the KLNF mixture membrane was unsuccessful due to brittleness of membrane, which broke upon applying pressure, however, sometimes we achieved a gel type membrane that usually stick to the surface of Cu electrodes and made it quite difficult to follow the calculation procedure.

The OLNF membrane shows improved conductivity than OLPONF i.e. ~17-18 Sm^{-1}. Although, this humble change in conductivity open up the vast door for the diverse possibilities to caper and tune it as required.

An attempt to measure the conductivity in a liquid phase (organic solvent) was done, in order to have an assurance of the direct influence of Nafion® on the conductivity. It seems to decrease up to ~10^{-4}, owing to the fact that Nafion®'s hydrophilic sulfonate groups could improve the solubility of quinone in water resulting ionization and production of aromatic anion. However, in the case of organic solvents the ionization - of quinone was restricted, which affected the conductivity. Usually, in the case of liquids, the conductivity shown within a mixture solution is proportional to its ion concentration.

Solid	Liquid
1.742×10^{-1} S.cm^{-2}	$1.79x10^{-4}$ S.cm^{-2}

To explain the loss of hydrophilic groups during the solution submersion, the conductivity has been noted after the wetting test, the OLPONF membranes were chosen on the basis of their higher mechanical intactness, the resistance and resistivity surprisingly increased after submersion, and there was a drop in conductivity that remain constant after applying pressure within the range of 1000-12000 Nm^{-2}(Figure. S2).

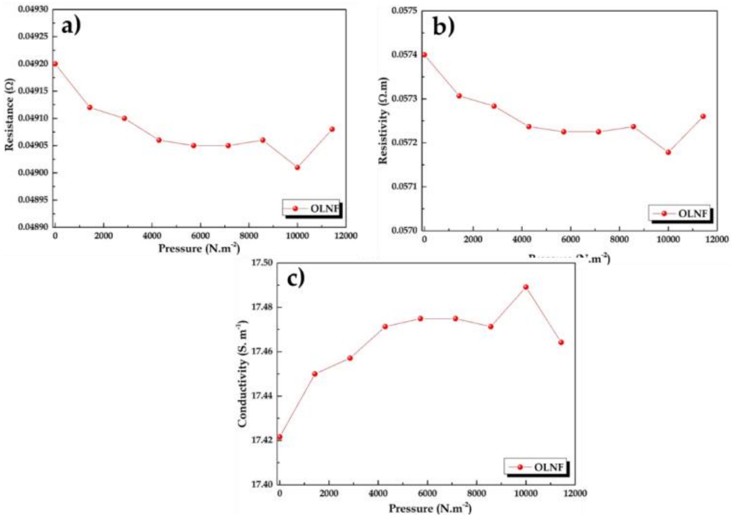

Figure. 7b: The variations of OLNF membranes in terms of a) Resistance, b) Resistivity and c) Conductivity with reference to applied pressure.

3.4. Cyclic Voltammetry (CV) measurements

Figure 8 shows the cyclic voltammetry (CV) profiles performed in the window of -1 to 1V potential range for the OLPONF and KLPONF as WE at different scanning rates to follow the redox activity corresponding to the quinone functionality of lignin (OL/KL) composite mixture. Meanwhile, the profiles for OLNF electrodes was measured at the scan rate of 20 mVs^{-1} for the comparison. Usually, the quinone moiety shows a sharp oxidation peak at ~0.6 V and an obvious reduction peak at ~0.4 V, however, the oxidation and reduction peak appears to shift to ~0.37-4V and ~0.3-0.6V vs. Ag/AgCl (Sat. KCl), respectively, possibly demonstrating the coordination interactions of ether oxygen of PEO and Nafion®'s carbonyl oxygen atoms of lignin, it might also be the hindrance due to SO_3H (sulfonic acid) group of Nafion®. The reversible faradic quinone/hydroquinone conversion reaction has been demonstrated in the schematic 1, where quinone functionality lose and gain 2 electrons/protons during the discharge and charge process.

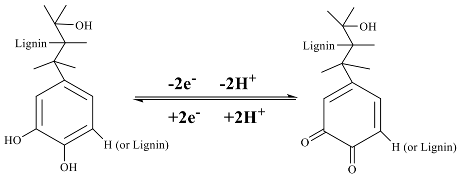

Schematic. 1: Redox activity of quinone/hydroquinone functionalities during charge-discharge process.

The sweep of different potential rate has been performed from 5-100 mVs^{-1}, however there wasn't any significant changes in the current rate, that could be due to the possibility of constant ion-adsorption dependent redox processes, this constant behaviour could be explained by the fact that the acidic electrolyte has been changed after each experiment, which could provide the constant ionic

mobility. The constant current profile could also be due to constant ratio of lignin in the sample electrodes.

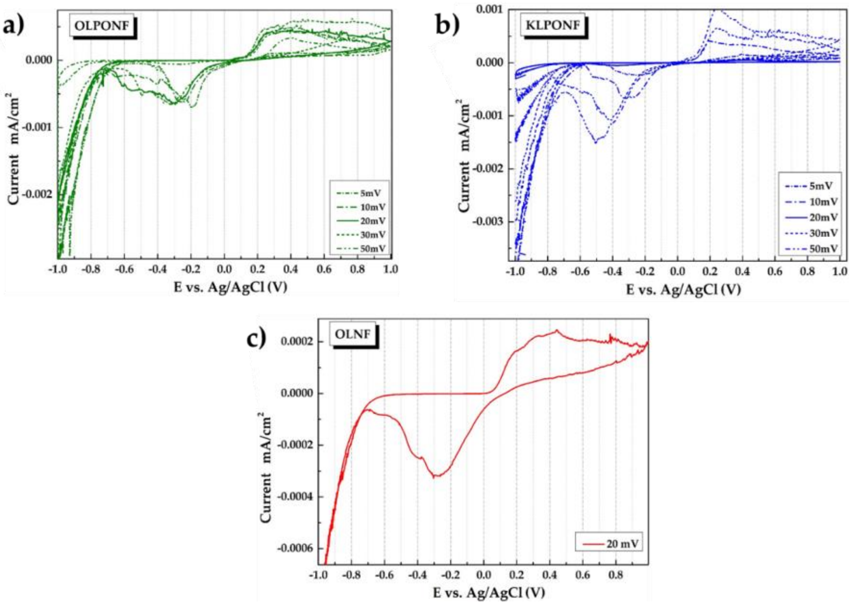

Figure. 8: Shows the four cyclic voltammetry (CV) profiles of the OLPO, OLNF, OLPONF and KLPONF electrodes performed in the window of -1V to 1V potential range at the scan rate of 20 mVs^{-1}.

The voltammogram of the samples with higher concentrations i.e. thicker gel like lignin composite shows very little redox activity, probably due to the insulating nature of lignin and the disintegration within the electrolytic solution, however, the low amount of ionic polymer wouldn't have any significant contribution to the electron storage.

3.5. Electrochemical Impedance Spectroscopy (EIS)

To further interpret the electrochemical performance, galvanostatic charging and discharging of the lignin composite mixtures was conducted. Though, the active mass loading of lignin has been maintained to 4 mg/mL. The charge−discharge 10 cycle curves at the current density of 100 mA.cm^{-2} between -1 and 1 V display different shapes compared to traditional capacitive ion storage, which seems quite incomprehensible. The curves look almost symmetrical indicating the charge storing ability of this composites. Excellent cycling stability of the electrode was obtained, which could be due to the unique structural features of the composites. Nafion® could act as a barrier and keep the lignin intact within the electrode, which could avoid the loss of active mass in the electrolyte and due to its ionic conductivity it could facilitate the charge transfer process by easing the efficient electron transport pathway. Additionally, the porous PEO polymer could work as a buffer, which provide the strain relaxation and volume change enabling the easy access of the electrolyte passage. Due to incomprehension and to be certain EIS measurements were conducted before and after the GCPL cycling measurements.

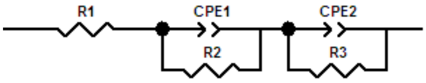

Schematic. 2: Series equivalent electric circuit model created on the basis of structural impedance behaviour of lignin based composite membranes.

Experimental impedance results are represented using Nyquist curves, where a non-linear adjustment techniques CNLS (complex nonlinear least square) was applied to a series equivalent circuit model (Schematic. 2). ECM series shows different components, where R1 represents the resistance of the assembly formed by electrolyte, working electrode and reference electrode. R2 is the interface resistance related to encapsulation of lignin within PEO and Nafion® structure. In order to represent the hydrophobic and heterogeneous structure of lignin mixture, CPE1 (constant phase element) have been used. CPE elements are able to reproduce the inhomogeneity related to the porous structure of the electrode, mass transfer phenomena or charge transfer reactions. The characteristics of the double layer are associated to the values of R3 (charge transfer resistance) and CPE2 (constant phase element).[54–56] Two capacitive arcs have been obtained, whose characteristic time constants correspond to two CPE-R pairs. The high frequency time constant, associated with the pair R2/CPE1 reflects the characteristics of the pore resistance and the low-frequency constant, reflects the characteristics of the load transfer resistance, associated with the pair R3/CPE2.The experimental impedance values fitted well with the series ECM.

To understand the physico-chemical processes within different time constants in the EIS spectra (Figure 9), the distribution of the relaxation times (DRT) of the experimental impedance data was calculated.[56–60]. Relaxation times obtained when fitting the impedance data to the ECM model, with respect to the times obtained when representing the same impedance data through the polarization processes, and (DRT). From the values of the different CPE1 and CPE2 the values of the capacitances can be obtained[61,62] as shown in Table S2 and S5.

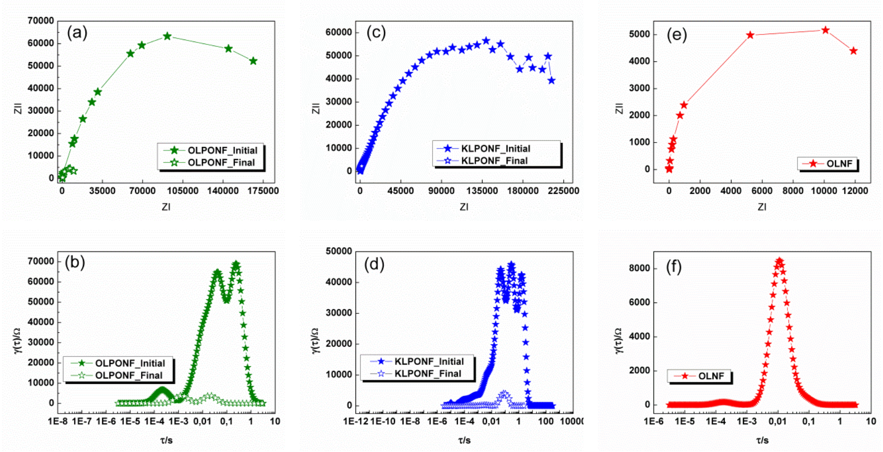

Figure. 9: Nyquist and DRT plots corresponding to the Impedance spectra of the; a-b) OLPONF and c-d) KLPONF, e-f) OLNF electrodes before and after the GCPL cycling measurements, respectively.

When comparing the time constants (τ) obtained through experimental and DRT calculations, it can be seen that the obtained results differs, the DRT values have been shown in Table S3. After cycling, KLPONF shows only a low frequency relaxation time, the high frequency time constant is absent within the DRT representation. The behavior of all lignin samples follows a decreasing pattern in the evolution of impedance. In the OLPONF and KLPONF samples this impedance drop is much more accentuated, decreasing much the interface resistance related to the encapsulation of lignin within the structure of PEO and Nafion®. The sample that best maintains its initial and final values and characteristics in a chemical environment is OLNF, possibly due to their structural integrity as shown by WU and SR% tests results.To validate the EIS data, the statistical analysis of repeated samples have been calculated (Figure S3), which reflects that the compound are highly electrochemically responsive depending on their composite mixture, surprisingly the KLPONF and OLPONF composites have shown more or less similar behavior in SD as well as in variance, however, the OLNF have been proven to show the highest distributive values.), which could be due to the behavioural changes of the biomass polymer (lignin) within the polymeric matrix i.e. difference between their intercross linkages, however, these outcomes need much strict understandings.

3.6 Perspectives and Limitations:

The main goal of this study is to develop lignin-based conductive composite materials and study their electrochemical properties for applications within electrochemical energy storage (EES) systems such as battery, fuel cell or supercapacitors. This study validates the idea of activation of lignin redox properties via simple modification in the polymeric matrix. However, lignin possesses diverse functionalities and its richness in aromaticity is yet to be explored, thus, our major goal is to provide a fully organic biomass conductive polymeric matrix via playing and tuning the functional groups of lignin using only simple and low-cost techniques that could open a door towards economical energy storage systems. Although, this study proposes an easy and inexpensive casting method to prepare composite membranes, however, the thickness control is the major limitations of this methodology that could lead to the probability of error despite following the exact steps and procedure. Thus, further studies will also be focused on the membrane casting optimization to establish internal and external validity of the result of unanticipated challenges that could emerged during the study.

5. Conclusions

Stable and functional lignin based composite (OL/KL-PONF) membranes were prepared by an easy mixing and solution casting method to be used as cheap electrodes. The present work shows consecutive characterization of the membranes to conclude the improved conductivity, the structural functionality and integrity. A strong ionic acid in mild reaction conditions was added to facilitate the pathway of oxidation and activation of the quinone reversible ($2e^-/2H^+$) redox cycling. The FTIR spectroscopy confirms the formation of composite mixture, where the shift in the vibration band of C-O-C and C=O stretching and appearance of Si-OH, Si-O-Si and CF_2 was observed due to incorporation of nafion®. Whereas, electrochemical processes and CV scans confirms the redox couple behavior and stability over repetitive cycling of the composite membranes. Further studies will continue on the in-depth understanding of lignin functionalities behavior along with exploration of potential modification routes and as Nafion® is an expensive polymer, the alternatives are needed to be studied in order to advance in the optimization of the composite membranes as well as of the electrochemical process.

Author Contributions: For research articles with several authors, a short paragraph specifying their individual contributions must be provided.

Marya Baloch has taken part in conceptualization, methodology, validation, formal analysis, investigation, data curation, writing—original draft preparation, writing—review and editing, and visualization.

Mikel Alberro Astarbe has taken part in formal analysis, data curation, writing—review and editing, and visualization.

Jalel Labidi has taken part in conceptualization, resources, writing—review and editing, visualization, supervision, project administration, and funding acquisition.

Funding: This research was funded by the Basque Government (project IT1008-16).

Acknowledgments: The authors thanks "Florencio Fernandez Marzo" and the laboratory of Chemical Engineering II at the department of Chemical and Environmental Engineering, School of Engineering of Gipuzkoa for their help and assistances with the electrochemical equipment.

Conflicts of Interest:

The authors declare no conflict of interest.

References

1. Chen, H.; Cong, T.N.; Yang, W.; Tan, C.; Li, Y.; Ding, Y. Progress in electrical energy storage system: A critical review. *Prog. Nat. Sci.* **2009**, *19*, 291–312, doi:10.1016/j.pnsc.2008.07.014.
2. Tong, Y.; Liang, J.; Liu, H.K.; Dou, S.X. Energy storage in Oceania. *Energy Storage Mater.* **2019**, doi:10.1016/j.ensm.2019.04.031.
3. Palomares, V.; Serras, P.; Villaluenga, I.; Hueso, K.B.; Carretero-González, J.; Rojo, T. Na-ion batteries, recent advances and present challenges to become low cost energy storage systems. *Energy Environ. Sci.* **2012**, *5*, 5884–5901, doi:10.1039/c2ee02781j.
4. Zhong, C.; Deng, Y.; Hu, W.; Qiao, J.; Zhang, L.; Zhang, J. A review of electrolyte materials and compositions for electrochemical supercapacitors. *Chem. Soc. Rev.* **2015**, *44*, 7484–7539, doi:10.1039/c5cs00303b.
5. Simon, P.; Gogotsi, Y. Materials for electrochemical capacitors. *Nat. Mater.* **2008**, *7*, 845–854.
6. Dehghani-Sanij, A.R.; Tharumalingam, E.; Dusseault, M.B.; Fraser, R. Study of energy storage systems and environmental challenges of batteries. *Renew. Sustain. Energy Rev.* **2019**, *104*, 192–208, doi:10.1016/j.rser.2019.01.023.
7. Ni, D.; Song, H.; Chen, Y.; Cai, K. Free-standing highly conducting PEDOT films for flexible thermoelectric generator. *Energy* **2019**, *170*, 53–61, doi:10.1016/j.energy.2018.12.124.
8. Popov, A.; Brasiunas, B.; Mikoliunaite, L.; Bagdziunas, G.; Ramanavicius, A.; Ramanaviciene, A. Comparative study of polyaniline (PANI), poly(3,4-ethylenedioxythiophene) (PEDOT) and PANI-PEDOT films electrochemically deposited on transparent indium thin oxide based electrodes. *Polymer (Guildf).* **2019**, *172*, 133–141, doi:10.1016/j.polymer.2019.03.059.
9. Chen, Q.; Wang, X.; Chen, F.; Zhang, N.; Ma, M. Extremely strong and tough polythiophene composite for flexible electronics. *Chem. Eng. J.* **2019**, *368*, 933–940, doi:10.1016/j.cej.2019.02.203.
10. Dong, J.; Lin, Y.; Zong, H.; Yang, H. Hierarchical $LiFe_5O_8$@PPy core-shell nanocomposites as electrode materials for supercapacitors. *Appl. Surf. Sci.* **2019**, *470*, 1043–1052, doi:10.1016/j.apsusc.2018.11.204.
11. Muniz, C.R.; Cunha, M.S. Effects of strong electric fields in a polyacetylene chain. *J. Phys. Chem. Solids* **2015**, *82*, 17–20, doi:10.1016/j.jpcs.2015.02.009.
12. Chen, Q.; Yin, Q.; Dong, A.; Gao, Y.; Qian, Y.; Wang, D.; Dong, M.; Shao, Q.; Liu, H.; Han, B.H.; et al. Metal complex hybrid composites based on fullerene-bearing porous polycarbazole for H_2, CO_2 and CH_4 uptake and heterogeneous hydrogenation catalysis. *Polymer (Guildf).* **2019**, *169*, 255–262, doi:10.1016/j.polymer.2019.02.056.
13. Gao, C.; Chen, G. Conducting polymer/carbon particle thermoelectric composites: Emerging

green energy materials. *Compos. Sci. Technol.* **2016**, *124*, 52–70, doi:10.1016/j.compscitech.2016.01.014.

14. Bae, J.; Park, J.Y.; Kwon, O.S.; Lee, C.S. Energy efficient capacitors based on graphene/conducting polymer hybrids. *J. Ind. Eng. Chem.* **2017**, *51*, 1–11, doi:10.1016/j.jiec.2017.02.023.

15. Zhu, H.; Luo, W.; Ciesielski, P.N.; Fang, Z.; Zhu, J.Y.; Henriksson, G.; Himmel, M.E.; Hu, L. Wood-Derived Materials for Green Electronics, Biological Devices, and Energy Applications. *Chem. Rev.* **2016**, *116*, 9305–9374, doi:10.1021/acs.chemrev.6b00225.

16. Silva, F.T.M.; Ataíde, C.H. Valorization of eucalyptus urograndis wood via carbonization: Product yields and characterization. *Energy* **2019**, *172*, 509–516, doi:10.1016/j.energy.2019.01.159.

17. Constant, S.; Wienk, H.L.J.; Frissen, A.E.; Peinder, P. De; Boelens, R.; Van Es, D.S.; Grisel, R.J.H.; Weckhuysen, B.M.; Huijgen, W.J.J.; Gosselink, R.J.A.; et al. New insights into the structure and composition of technical lignins: A comparative characterisation study. *Green Chem.* **2016**, *18*, 2651–2665, doi:10.1039/c5gc03043a.

18. Hirai, N.; Tanaka, T.; Kubo, S.; Ikeda, T.; Magara, K.; Ban, I.; Shiota, M. Density and hardness of negative pastes of lead-acid batteries containing organic additives with or without quinone structure. *J. Power Sources* **2006**, *158*, 1106–1109, doi:10.1016/j.jpowsour.2006.02.002.

19. Jablonsky, M.; Andrea, S.; Haz, A.; Ľudmila, H.; Michal, J.; Andrea, Š.; Aleš, H. *Lignin, potential products and their market value*;

20. Amezcua-Allieri, M.A.; Aburto, J. Conversion of Lignin to Heat and Power, Chemicals or Fuels into the Transition Energy Strategy. *Lignin - Trends Appl.* **2018**, doi:10.5772/intechopen.71211.

21. Xia, K.; Ouyang, Q.; Chen, Y.; Wang, X.; Qian, X.; Wang, L. Preparation and Characterization of Lignosulfonate-Acrylonitrile Copolymer as a Novel Carbon Fiber Precursor. *ACS Sustain. Chem. Eng.* **2016**, *4*, 159–168, doi:10.1021/acssuschemeng.5b01442.

22. Lu, H.; Zhao, X.S. Biomass-derived carbon electrode materials for supercapacitors. *Sustain. Energy Fuels* **2017**, *1*, 1265–1281, doi:10.1039/C7SE00099E.

23. Fang, Z.; Fan, L.; Grace, J.R.; Ni, Y.; Scott, N.R.; Smith, R.L. Biofuels and Biorefi neries. *6*.

24. Tenhaeff, W.E.; Rios, O.; More, K.; McGuire, M.A. Highly robust lithium ion battery anodes from lignin: An abundant, renewable, and low-cost material. *Adv. Funct. Mater.* **2014**, *24*, 86–94, doi:10.1002/adfm.201301420.

25. Lu, H.; Cornell, A.; Alvarado, F.; Behm, M.; Leijonmarck, S.; Li, J.; Tomani, P.; Lindbergh, G. Lignin as a binder material for eco-friendly Li-ion batteries. *Materials (Basel).* **2016**, *9*, 1–17, doi:10.3390/ma9030127.

26. Nirmale, T.C.; Kale, B.B.; Varma, A.J. A review on cellulose and lignin based binders and electrodes: Small steps towards a sustainable lithium ion battery. *Int. J. Biol. Macromol.* **2017**, *103*, 1032–1043, doi:10.1016/j.ijbiomac.2017.05.155.

27. Mukhopadhyay, A.; Hamel, J.; Katahira, R.; Zhu, H. Metal-Free Aqueous Flow Battery with Novel Ultrafiltered Lignin as Electrolyte. *ACS Sustain. Chem. Eng.* **2018**, *6*, 5394–5400, doi:10.1021/acssuschemeng.8b00221.

28. Hill, C.A.S. The Use of Timber in the Twenty-first Century. *Wood Modif.* **2006**, 1–18, doi:10.1002/0470021748.ch1.

29. Matrakova, M.; Rogachev, T.; Pavlov, D.; Myrvold, B.O. Influence of phenolic group content in lignin expanders on the performance of negative lead-acid battery plates. *J. Power Sources* **2003**, *113*, 345–354, doi:10.1016/S0378-7753(02)00547-5.

30. Gnedenkov, S. V.; Opra, D.P.; Zemnukhova, L.A.; Sinebryukhov, S.L.; Kedrinskii, I.A.; Patrusheva, O. V.; Sergienko, V.I. Electrochemical performance of Klason lignin as a low-cost cathode-active material for primary lithium battery. *J. Energy Chem.* **2015**, *24*, 346–352, doi:10.1016/S2095-4956(15)60321-7.

31. Wu, X.; Jiang, J.; Wang, C.; Liu, J.; Pu, Y.; Ragauskas, A.; Li, S.; Yang, B. Lignin-derived electrochemical energy materials and systems. *Biofuels, Bioprod. Biorefining* **2020**, *14*, 650–672, doi:10.1002/bbb.2083.

32. Liu, B.; Huang, Y.; Cao, H.; Song, A.; Lin, Y.; Wang, M.; Li, X. A high-performance and environment-friendly gel polymer electrolyte for lithium ion battery based on composited lignin membrane. *J. Solid State Electrochem.* **2018**, *22*, 807–816, doi:10.1007/s10008-017-3814-x.

33. Chaleawlert-umpon, S.; Berthold, T.; Wang, X.; Antonietti, M.; Liedel, C. Kraft Lignin as Electrode Material for Sustainable Electrochemical Energy Storage. *Adv. Mater. Interfaces* **2017**, *4*, 1–7, doi:10.1002/admi.201700698.

34. Casado, N.; Hilder, M.; Pozo-Gonzalo, C.; Forsyth, M.; Mecerreyes, D. Electrochemical Behavior of PEDOT/Lignin in Ionic Liquid Electrolytes: Suitable Cathode/Electrolyte System for Sodium Batteries. *ChemSusChem* **2017**, *10*, 1783–1791, doi:10.1002/cssc.201700012.

35. Wang, S.; Zhang, L.L.; Wang, A.; Liu, X.; Chen, J.; Wang, Z.; Zeng, Q.; Zhou, H.H.; Jiang, X.; Zhang, L.L. Polymer-Laden Composite Lignin-Based Electrolyte Membrane for High-Performance Lithium Batteries. *ACS Sustain. Chem. Eng.* **2018**, *6*, 14460–14469, doi:10.1021/acssuschemeng.8b03117.

36. Gnedenkov, S.V.V. V; Sinebryukhov, S.L.L.L.; Opra, D.P.P.P.; Zemnukhova, L.A.A.A.; Tsvetnikov, A.K.K.; Minaev, A.N.N.; Sokolov, A.A.A.; Sergienko, V.I.I. Electrochemistry of Klason Lignin. *Procedia Chem.* **2014**, *11*, 96–100, doi:10.1016/j.proche.2014.11.018.

37. Gnedenkov, S. V.; Opra, D.P.; Sinebryukhov, S.L.; Tsvetnikov, A.K.; Ustinov, A.Y.; Sergienko,

V.I. Hydrolysis lignin: Electrochemical properties of the organic cathode material for primary lithium battery. *J. Ind. Eng. Chem.* **2014**, *20*, 903–910, doi:10.1016/j.jiec.2013.06.021.

38. Ye, J.; Lou, X.; Wu, C.; Wu, S.; Ding, M.; Sun, L.; Jia, C.; Ding, M. Ion Selectivity and Stability Enhancement of SPEEK / Lignin Membrane for Vanadium Redox Flow Battery : The Degree of Sulfonation Effect. *Front. Chem.* **2019**, *6*, 1–9, doi:10.3389/fchem.2018.00549.

39. Ye, J.; Cheng, Y.; Sun, L.; Ding, M.; Wu, C.; Yuan, D.; Zhao, X.; Xiang, C.; Jia, C. A green SPEEK/lignin composite membrane with high ion selectivity for vanadium redox flow battery. *J. Memb. Sci.* **2019**, *572*, 110–118, doi:10.1016/j.memsci.2018.11.009.

40. Admassie, S.; Ajjan, F.N.; Elfwing, A.; Inganäs, O. Biopolymer hybrid electrodes for scalable electricity storage. *Mater. Horizons* **2016**, *3*, 174–185, doi:10.1039/c5mh00261c.

41. Rębiś, T.; Nilsson, T.Y.; Inganäs, O. Hybrid materials from organic electronic conductors and synthetic-lignin models for charge storage applications. *J. Mater. Chem. A* **2016**, *4*, 1931–1940, doi:10.1039/c5ta06821e.

42. Milczarek, G. Lignosulfonate-modified electrodes: Electrochemical properties and electrocatalysis of NADH oxidation. *Langmuir* **2009**, *25*, 10345–10353, doi:10.1021/la9008575.

43. Milczarek, G. Preparation and characterization of a lignin modified electrode. *Electroanalysis* **2007**, *19*, 1411–1414, doi:10.1002/elan.200703870.

44. Lota, G.; Milczarek, G. The effect of lignosulfonates as electrolyte additives on the electrochemical performance of supercapacitors. *Electrochem. commun.* **2011**, *13*, 470–473, doi:10.1016/j.elecom.2011.02.023.

45. Zhu, J.; Yan, C.; Zhang, X.; Yang, C.; Jiang, M.; Zhang, X. A sustainable platform of lignin: From bioresources to materials and their applications in rechargeable batteries and supercapacitors. *Prog. Energy Combust. Sci.* **2020**, *76*, 100788, doi:10.1016/j.pecs.2019.100788.

46. Becker, J.; Wittmann, C. A field of dreams: Lignin valorization into chemicals, materials, fuels, and health-care products. *Biotechnol. Adv.* **2019**, *37*, 107360, doi:10.1016/j.biotechadv.2019.02.016.

47. Editors: Hakeem, Khalid Rehman, Jawaid, Mohammad, Alothman, O. *Agricultural Biomass Based Potential Materials*; 2015; ISBN 9783319138473.

48. Obydenkova, S. V.; Kouris, P.D.; Hensen, E.J.M.M.; Heeres, H.J.; Boot, M.D. Environmental economics of lignin derived transport fuels. *Bioresour. Technol.* **2017**, *243*, 589–599, doi:10.1016/j.biortech.2017.06.157.

49. Ragauskas, A.J.; Beckham, G.T.; Biddy, M.J.; Chandra, R.; Chen, F.; Davis, M.F.; Davison, B.H.; Dixon, R.A.; Gilna, P.; Keller, M.; et al. Lignin valorization: Improving lignin processing in the biorefinery. *Science (80-.).* **2014**, *344*, doi:10.1126/science.1246843.

50. Ding, R.; Wu, H.; Thunga, M.; Bowler, N.; Kessler, M.R. Processing and characterization of low-cost electrospun carbon fibers from organosolv lignin/polyacrylonitrile blends. *Carbon N. Y.* **2016**, *100*, 126–136, doi:10.1016/j.carbon.2015.12.078.

51. Fodil Cherif, M.; Trache, D.; Brosse, N.; Benaliouche, F.; Tarchoun, A.F. Comparison of the Physicochemical Properties and Thermal Stability of Organosolv and Kraft Lignins from Hardwood and Softwood Biomass for Their Potential Valorization. *Waste and Biomass Valorization* **2020**, *11*, 6541–6553, doi:10.1007/s12649-020-00955-0.

52. Liu, G.; Shi, H.; Ping, Q.; Zhou, J.; Zhang, J.; Li, N.; Niu, M.; Fatehi, P.; Xiao, H.; Ni, Y. Complex Formation of PEO and Lignin in Prehydrolysis Liquor and its Enhancing Effect on Lignin Removal. *BioResources* **2013**, *8*, 4004–4015, doi:10.15376/biores.8.3.4004-4015.

53. Kubo, S.; Kadla, J.F. Kraft lignin/poly(ethylene oxide) blends: Effect of lignin structure on miscibility and hydrogen bonding. *J. Appl. Polym. Sci.* **2005**, *98*, 1437–1444, doi:10.1002/app.22245.

54. Chen, T.; Zhang, Q.; Xu, J.; Pan, J.; Cheng, Y.T. Binder-free lithium ion battery electrodes made of silicon and pyrolized lignin†. *RSC Adv.* **2016**, *6*, 29308–29313, doi:10.1039/c6ra03001g.

55. Stergiou, D. V.; Veltsistas, P.G.; Prodromidis, M.I. An electrochemical study of lignin films degradation: Proof-of-concept for an impedimetric ozone sensor. *Sensors Actuators, B Chem.* **2008**, *129*, 903–908, doi:10.1016/j.snb.2007.10.001.

56. Illig, J.; Chrobak, T.; Klotz, D.; Ivers-Tiffée, E. Evaluation of the Rate Determining Processes for LiFePO 4 as Cathode Material in Lithium-Ion-Batteries . *ECS Trans.* **2019**, *33*, 3–15, doi:10.1149/1.3564865.

57. https://sites.google.com/site/drttools/.

58. Ciucci, F.; Chen, C. Analysis of electrochemical impedance spectroscopy data using the distribution of relaxation times: A Bayesian and hierarchical Bayesian approach. *Electrochim. Acta* **2015**, *167*, 439–454, doi:10.1016/j.electacta.2015.03.123.

59. Schmidt, J.P.; Chrobak, T.; Ender, M.; Illig, J.; Klotz, D.; Ivers-Tiffée, E. Studies on LiFePO4 as cathode material using impedance spectroscopy. *J. Power Sources* **2011**, *196*, 5342–5348, doi:10.1016/j.jpowsour.2010.09.121.

60. Li, X.; Ahmadi, M.; Collins, L.; Kalinin, S. V. Deconvolving distribution of relaxation times, resistances and inductance from electrochemical impedance spectroscopy via statistical model selection: Exploiting structural-sparsity regularization and data-driven parameter tuning. *Electrochim. Acta* **2019**, *313*, 570–583, doi:10.1016/j.electacta.2019.05.010.

61. Li, Q.; Thangadurai, V. Synthesis, Structure and Electrical Properties of Mo-doped CeO2–Materials for SOFCs. *Fuel Cells* **2009**, *9*, 684–698, doi:https://doi.org/10.1002/fuce.200900044.

62. Li, Q.; Xia, T.; Liu, X.D.; Ma, X.F.; Meng, J.; Cao, X.Q. Fast densification and electrical

conductivity of yttria-stabilized zirconia nanoceramics. *Mater. Sci. Eng. B Solid-State Mater. Adv. Technol.* **2007**, *138*, 78–83, doi:10.1016/j.mseb.2006.12.012.

Publisher's Note: MDPI stays neutral with regard to jurisdictional claims in published maps and institutional affiliations.

© 2020 by the authors. Submitted for possible open access publication under the terms and conditions of the Creative Commons Attribution (CC BY) license (http://creativecommons.org/licenses/by/4.0/).
